# Effects and Safe Inclusion of Narbonne Vetch (*Vicia narbonensis*) in Rainbow Trout (*Oncorhynchus mykiss*) Diets: Towards a More Sustainable Aquaculture

**DOI:** 10.3390/ani10112175

**Published:** 2020-11-21

**Authors:** Cristina Tomás-Almenar, Francisco Javier Toledo-Solís, Ana M. Larrán, Eduardo de Mercado, Francisco Javier Alarcón, Daniel Rico, Ana Belén Martín-Diana, Ignacio Fernández

**Affiliations:** 1 Aquaculture Research Center, Agro-Technological Institute of Castilla y León (ITACyL), Ctra. Arévalo, 40196 Zamarramala, Segovia, Spain; tomalmcr@itacyl.es (C.T.-A.); ita-largaran@itacyl.es (A.M.L.); 2 Consejo Nacional de Ciencia y Tecnología (CONACYT), Av. Insurgentes Sur 1582, Col. Crédito 6 Constructor, Alcaldía Benito Juárez, Mexico City C.P. 03940, Mexico; fj.toledos@gmail.com; 3 Department Biology and Geology, Ceimar-University of Almería, 04120 Almería, Spain; falarcon@ual.es; 4 Pig Research Center, Agro-Technological Institute of Castilla y León (ITACyL), Ctra. Riaza Toro S-N, 40353 Hontalbilla, Segovia, Spain; eduardo.mercado@inia.es; 5 Agro-Technological Institute of Castilla y León (ITACyL), Ctra. de Burgos Km.119, Finca Zamadueñas, 47071 Valladolid, Spain; ricbarda@itacyl.es (D.R.); mardiaan@itacyl.es (A.B.M.-D.)

**Keywords:** antinutritional factors, legume, local crops, vegetable meal

## Abstract

**Simple Summary:**

Aquaculture’s sustainability deeply relies on the identification and inclusion of alternative raw materials rather than continuing with the use of finite resources such as fish meal and fish oil. Different alternative raw materials have been tested, with the meal from terrestrial vegetable species being one of the main substitution candidates for fish meal. This study evaluated the effects of the inclusion (at 0%, 10% and 30%) of Narbonne vetch (*Vicia narbonensis*) meal in rainbow trout (*Oncorhynchus mykiss*) diets as a first attempt to evaluate its safety as a suitable alternative raw material. High inclusion (30%) of Narbonne vetch led to a reduced final fish size and weight. This seemed to be due to severe histopathological alterations in the proximal intestine that might alter the capacity of the fish to efficiently use the nutrients supplied by the diet. In contrast, a 10% inclusion did not affect fish growth nor digestive system and/or the nutritional value of the fish fillet. Although further research efforts might be required to increase the utilization of alternative raw materials locally produced like Narbonne vetch, present results show Narbonne vetch might reduce carbon footprint in European aquaculture and the dependency on other alternative raw materials such as soybean (*Glycine max*) imported from third countries.

**Abstract:**

Aquaculture’s sustainability deeply relies on the identification and inclusion of alternative raw materials. Although meals from insects and/or byproducts from different industries are being recently tested, the meal from terrestrial vegetable species is still the main substitution candidate for fish meal. Here the effects of 0% (Control), 10% (A10) and 30% (A30) inclusion of Narbonne vetch (*Vicia narbonensis*; ZV-156 strain) meal in rainbow trout (*Oncorhynchus mykiss*) diets was assessed in a 63-day feeding trial by means of growth performance, histopathological, nutritional value of the fish fillet and blood biochemistry analyses. A dose-response trial was conducted in triplicate with 25 rainbow trout juveniles (20 g average body weight) per 500 L tank. Narbonne vetch meal decreased total intestine protease activity in vitro (from 26.81% to 48% inhibition), although high temperature partially inhibited the action of antinutritional factors (ANFs). No differences in fish growth performance and no severe histopathological alterations on the proximal intestine were observed between 10% Narbonne vetch inclusion and Control groups. In contrast, high inclusion (30%) of Narbonne vetch led to poor growth performance (30% reduction on final growth) and severe histopathological alterations (e.g., loss of brush border integrity, high number of villi fusion, reduced goblet cells density as well as reduced width of submucosa, muscular and serosa layers). Furthermore, while the A30 diet decreased docosahexaenoic fatty acid (FA) content in fish fillets, the A10 diet improved monounsaturated FA content when compared to that of the Control group. No altered levels of cholesterol, glucose or triglycerides in blood plasma and/or histopathological effects on the liver were observed among fish fed the different experimental diets. Although further research efforts (e.g., identifying potential enzymatic treatments to decrease the action of ANFs from Narbonne vetch meal) might be required, present results show that a low inclusion (10%) of Narbonne vetch in rainbow trout diets is possible. The inclusion of locally produced legumes such a Narbonne vetch might be an interesting approach to reduce carbon footprint in European aquaculture and the dependency on other alternative raw materials such as soybean (*Glycine max*) imported from third countries.

## 1. Introduction

The production of aquatic organisms has (from 35.6 to 82.1 M t between 2000 and 2018), and still needs to be, increased in order to cope with the human population demands [1]. Since fish meal (FM) and oil—the golden standard raw materials fulfilling the nutritional requirements of fish [2]—are limited sources, an enormous research effort to identify and implement new and more sustainable alternative raw materials in aquafeeds has been conducted in the last decade [3,4]. Although new alternatives have been recently explored [5,6], protein sources from terrestrial vegetables are still the major candidates for substitution FM [7,8]. Nevertheless, several issues have limited the use of these alternative protein sources, such as the presence of antinutritional factors (ANFs), phytoestrogens and non-soluble carbohydrates, the lower digestibility of crude protein and bioavailability of minerals and/or the deficiencies in some amino acids, among other factors [9,10,11]. Soybean (*Glycine max*) meal (SBM) stands as the most used alternative raw material to substitute FM (up to 20–40%), among the other vegetable protein sources considered [7,12,13]. Most of SBM is produced in the USA, Argentina and Brazil and thus, European aquaculture largely depends on importation from third countries [14]. As a consequence, there is a large interest in identifying alternative vegetable protein sources that are locally produced and can be used in feedstuffs for fish species, in order to reduce EU dependency and carbon footprint.

Narbonne vetch (*Vicia narbonensis*) is another legume such as soybean with a great potential to be used in feedstuffs. It is typically cultivated in the Mediterranean region, growing in dry and/or semiarid habitats [15,16], requires low maintenance, is resistant to plagues, has a rapid growth in winter and renders approximately 1.50 t of grains per ha [17,18]. Furthermore, Narbonne vetch seeds have 28% crude protein and a quite balanced amino acid profile [19]. It is considered as a low-cost protein source for animal feeds in order to reduce SBM dependency [20,21]. Nevertheless, as other species of the genus *Vicia*, it contains different ANFs such as *γ*-glutamyl-S-ethenyl cysteine (GEC) which particularly reduce the feed intake in farmed animals. Narbonne vetch inclusion has been tested in diets for pigs [22,23], poultry [24] and bovine [25] species. Until now, only one study reported the effects of Narbonne vetch inclusion on fish growth performance. In this sense, Buyukcapar and co-workers [26] explored the use (up to 40% inclusion) of Narbonne vetch seeds meal in diets for tilapia (*Oreochromis niloticus*), and reported a reduced feed intake, growth performance and lower lipid content in fillets of fingerlings fed diets with inclusions of Narbonne vetch meal above 20%.

Rainbow trout (*Oncorhynchus mykiss*) is one of the most largely farmed fish species worldwide (>848,000 t y^−1^), the biggest in the European (185,316 t) region and the second species produced in Spain (18,955 t vs. the 27,335 t of European seabass (*Dicentrarchus labrax*)) [1,27]. In this sense, a large research effort has been done in order to implement alternative raw materials in rainbow trout diets, from vegetable oils [28,29,30] to a diverse set of meals from different sources (e.g., soybean, insects, algae, animal by-products, microorganisms) [31,32,33,34,35,36,37,38] in order to substitute FM and FO. Here, the inclusion of Narbonne vetch (ZV-156 strain) meal in diets for rainbow trout juveniles was explored, assessing the effects on growth performance, but also determining the impact of ANFs on total intestinal protease activity in vitro, and the physiological status of fish based on the histopathological analysis of the digestive system, blood biochemistry and the nutritional value of the fillet. The present study aims to identify the factors limiting the inclusion of Narbonne vetch meal in fish diets towards a more sustainable and independent European aquaculture.

## 2. Materials and Methods

### 2.1. Narbonne Vetch Seeds

Seeds of Narbonne vetch strain ZV-156 harvested in 2016 were obtained from the Crop Production Department (ITACyL). The seeds were ground and sieved through a 0.7-mm sieve prior to their inclusion in feed formulation. The proximate composition of Narbonne vetch meal, the amino acid profile and the content of GEC are shown in Table 1.

The amino acid analysis profile of Narbonne vetch meal was performed using 20 mg of meal. After hydrolysis with 1 mL of 6 N HCl for 24 h at 110 °C, samples were neutralized with NaOH 6.5 N, and diluted ten times with loading buffer pH 2.2 (80-2037-67, Biochrom, Cambridge, UK). The determination was performed by ion-exchange liquid chromatography and post-column continuous reaction with ninhydrin (Biochrom 30+, Cambridge, UK) to provide qualitative and quantitative compositional analysis. Norleucine was used as the internal standard.

### 2.2. Experimental Diets

Diets were formulated containing different inclusion levels of Narbonne vetch meal: 0% (Control diet), 10% and 30% (A10 and A30 diets, respectively). Diets were adjusted to reach isoproteic and isolipidic values, and supplemented with methionine to fulfill the nutritional requirements of rainbow trout (Table 2). Feeds were manufactured by LifeBioencapsulation S.L. (Almeria, Spain), and appropriated size pellets (2–3 mm) were extruded.

### 2.3. In Vitro Inhibition of Rainbow Trout Digestive Protease Activity by Narbonne Vetch Meal and Experimental Feeds

#### 2.3.1. Preparation of Enzyme Extracts

The digestive tracts of 10 rainbow trout specimens (approx. 30 g mean body weight) were dissected into intestines and pyloric ceca at 4 °C. Crude enzyme extracts were obtained by homogenizing tissues with an UltraTurrax T25 basic (IKA©-Werke, Staufen, Germany) in distilled water to a final concentration of 0.5 g/mL. The tissues were further disrupted to release the enzymes by sonication (3 cycles, 20 s/cycle) over ice. The homogenate was centrifuged at 16,000× *g* for 15 min at 4 °C, and the supernatant was used as enzyme extract (EE) and stored at −20 °C until assayed.

#### 2.3.2. Inhibition of Intestinal Protease Activity by Narbonne Vetch Meal and Experimental Diets

Protease inhibition assay was done according to [39]. An aqueous solution of Narbonne vetch meal or experimental feeds was prepared at 0.1 g/mL and maintained in agitation for 24 h at 4 °C before centrifugation at 16,000× *g* for 15 min at 4 °C. The supernatant was used as inhibitor extract (IE). Increasing quantities of IE (50, 100, 200, 300 and 400 µg of Narbonne vetch meal, and 165, 330, 495, 660 and 990 µg of experimental feeds) were mixed with a fixed volume of EE (distilled water was added to reach equal volumes), and incubated for 1 h at 4 °C. After incubation, 0.5 mL of Tris-HCl buffer (pH 9.0) was added to 10 µL of IE-EE, and the reaction was started after adding 0.5 mL of 1% casein. The mixtures were incubated at 37 °C for 1 h and the reaction was stopped by adding 0.5 mL of 20% trichloroacetic acid (TCA) and cooled at −20 °C for 15 min. After centrifugation at 16,000× *g* for 15 min at 4 °C, the absorbance of supernatant was recorded at 280 nm (Genesis 30, Thermo Fisher Scientific, Alcobendas, Spain). The total protease activity was transformed in units per mL of EE (U/mL), and the specific activity of the extracts were also determined and expressed as U/g of tissue. The percentage of inhibition was calculated respect to a control assay where the IE of Narbonne vetch meal was substituted by an equal volume of distilled water. The fish enzyme solution was standardized to produce an absorbance at 280 nm of 0.5 in the control assay. Values of inhibition were expressed as a percentage of residual activity, considering the activity of the control as 100%. Controls were made replacing the test substance (Narbonne vetch or experimental feed extracts) by distilled water. The inhibition of total protease activity was performed in Narbonne vetch meal and experimental diets nontreated and preheated at 60, 80 and 100 °C for 30 min (simulating the extrusion process). The inhibition of total protease activity in the Narbonne vetch meal under different temperatures and the experimental diets was tested in triplicate.

### 2.4. Ethical Statement

All experiments complied with the Animal Research: Reporting of In Vivo Experiments (ARRIVE) guidelines [40] and were performed according to 2010/63/EU of the European Parliament and Council, guideline 86/609/EU of the European Union Council and Spanish legislation (RD53/2013), in order to warrant an ethical animal experimentation as well as fish welfare. The persons involved in the experiments are qualified to handle animals for experimentation according to Orden ECC/566/2015 from Spanish legislation. All procedures were previously approved by the Bioethical Committee of ITACyL in order to fulfill the administrative requirements prior to conducting the planned research (approval number: 2018/31/CEEA).

### 2.5. Experimental Design and Rearing Conditions

A total of 400 all-female rainbow trout juveniles of 20 g (approx. mean body weight) were obtained from a commercial farm “Piscifactoría Fuente del Campillo” located in Guadalajara (Spain), and shipped to the experimental facilities of the Aquaculture Research Center (ITACyL, Segovia, Spain). Fish were acclimatized for 15 days before growth trial, after which they were randomly allocated into 9 cylindrical fiberglass tanks (500 L) connected to a recirculating aquaculture system. Twenty-five animals with a mean weight of 26.8 ± 0.7 g and a total length (TL) of 13.4 ± 0.1 cm were allocated in each tank, and experimental diets were tested in triplicate. Fish were hand-fed to apparent satiation once (between 8:00 and 9:00 h) a day (until a maximum of 3% daily feed intake) for 63 days.

During the growth trial, water temperature was maintained at 15 ± 1 °C, containing > 7 ± 1 mg/L of dissolved oxygen, and under a 12:12-h light:dark photoperiod cycle. The ammonium and nitrite water concentrations were daily monitored to keep them below toxic values.

### 2.6. Fish Sampling and Growth Performance Assessment

At the end of the trial, 3 fish from each tank were also randomly sampled and sacrificed with an overdose of MS222 (300 mg/mL). In this case, fish were dissected to collect blood, liver, proximal intestine as well as fillet samples for their corresponding analyses. Along the experiment, growth, in terms of weight and total length (TL), was monitored all 21 days in order to adjust daily feed ration. In this sense, fish were slightly anesthetized with MS-222 (180 mg/mL). TL was assessed using a graduated ichthyometer (±0.1 mm) and wet weight with a GRAM S3R-6KD balance (±0.1 g). Every day, mortality and feed intake in each tank were recorded. Feces were collected during the last two weeks for apparent digestibility analysis by a modified Guelph method [41]. Feces from each experimental tank were stored at −80 °C until analysis.

Growth performance indexes were calculated according to the following equations:

WG (weight gain, %) = [(FBW − IBW)/IBW] × 100 FBW and IBW are final body weight (g) and initial body weight (g), respectively.(1)

SGR (specific growth rate, %/day) = [(ln FBW − ln IBW)/days] × 100(2)

FCR (feed conversion ratio) = [total feed intake (g)/WG (g)](3)

CF (condition factor) = [weight (g)/TL^3^ (cm)] × 100 (4)

HSI (hepato-somatic index, %) = [wet liver weight (g)/FBW] × 100(5)

VSI (viscero-somatic index, %) = [wet visceral weight (g)/FBW] × 100(6)

### 2.7. Serum Biochemical Assays

Blood samples were taken from the caudal vein using 1-mL, plastic syringes coated with Lithium Heparin (L-Heparin) as anticoagulant, and transferred to a 1-mL tube with L-Heparin (MiniCollect^®^, Kremsmünster, Austria). The plasma was obtained by centrifugation at 6600× *g* for 20 min at 4 °C and stored at −80 °C until analysis.

All biochemical (triglycerides, glucose and cholesterol) analyses in plasma were performed with colorimetric assay kits according to the manufacturer instructions (Bio-Science Medical S.L., Madrid, Spain). Absorbance was measured in triplicate in 96-well microplates using a microplate reader (ELx800TM; BioTek Instruments, Inc., Winooski, VT, USA).

### 2.8. Histopathological Analysis

At the end of the experiment, dissected liver, proximal and distal intestine tissues of rainbow trout were fixed by immersion in 4% buffered paraformaldehyde (pH 7.4) during 24 h at room temperature. Dehydration of fixed samples was performed by transferring samples in a sequential series of graded alcohol solutions (25%, 50%, 75% and 100%). Samples were embedded in paraffin blocks, sectioned (3–5 µm) and stained with Haematoxylin-Eosin and Alcian blue (AB, pH = 2.5) periodic acid-Schiff (PAS) solutions to characterize liver and intestine histomorphology, as well as to identify and quantify goblet cell’s density in intestinal sections. All procedures were performed as described by [42,43,44].

Mounted sections were photographed with an Olympus EP50 camera at an Olympus CX31 microscope while image analysis was performed with Image J software. The following parameters were assessed at the proximal intestine of 3 fish per experimental tank: height of villi, integrity of brush border membrane, supranuclear vacuolization degree, localization of nucleus in the enterocytes as well as enterocyte’s height, submucosa, muscularia and serosa layer width and density of goblet cells. Hepatocytes shrinkage were evaluated by percentage of mean surface not covered by these cells in three liver subsections of each histological section. For any evaluated parameter, at least 3 measurements per section were performed.

### 2.9. Chemical Analyses

The apparent digestibility of the protein was determined using acid-insoluble ash as a marker in feces [45], and calculated as follows:

ADCprot (apparent digestibility coefficient of protein) = 100 − [(marker in diet/marker in feces) × (% protein in feces/% protein in diet) × 100](7)

Moisture and protein content in the different matrix were determined according to official Association of Official Agricultural Chemists (AOAC) procedures [46] and the Official Journal of the European Union [47]. The moisture was calculated by drying samples at 105 °C for 24 h until constant weight. Protein content were analyzed by the Kjeldahl method (N × 6.25), fat by dichloromethane extraction (Soxhlet) and ash content by heating the residue from the moisture determination in a muffle furnace at 550 °C for 24 h. All parameters were expressed in percentage in relation to dry matter. Analyses were conducted in triplicate.

Fatty acid (FA) profile was determined for fish fillets in triplicate. The Bligh and Dyer method was used for lipid extraction [48]. The lipid-containing chloroform phase was separated and evaporated. The remaining phase was dissolved in 1 mL of hexane, and a methylated procedure was carried out by adding 100 μL of 0.5 M methanolic KOH and leaving the reaction for 10 min at room temperature. The upper layer was transferred to a 2-mL vial. Analysis of FA methyl esters (FAME) was carried out on a gas chromatograph Agilent 7890A (Agilent Technologies, Santa Clara, CA, USA) and a flame ionization detector. For the analysis, the method was run at 50 °C to 200 °C for the first 7 min at a rate of 3 °C min^−1^ and held for 26 min. Injector and detector temperatures were 250 °C and 280 °C, respectively. After, 1 μL of the hexane extract was injected in split mode (ratio 25:1), and FAMEs were identified by comparison of retention times with those of the 37 FAMEs standard mix (Supelco, Sigma-Aldrich, St. Louis, MO, USA). FA profile was expressed in percentage.

### 2.10. Statistical Analysis

Results are given as mean values ± standard deviations. All data were previously checked for normality (Kolmogorov–Smirnov test) and homoscedasticity of variance (Bartlett’s test). Results were compared by means of one-way ANOVA and when significant differences were detected the Tukey multiple-comparison test was used to detect differences among experimental groups. The level of significance was set at *p* < 0.05. All the statistical analyses were conducted using GraphPad Prism 5.0 (GraphPad Software, Inc., San Diego, CA, USA).

## 3. Results

### 3.1. Inhibition of Intestinal Protease Activity by Narbonne Vetch Meal and Experimental Diets

Protease activity of rainbow trout was directly inhibited by the increased presence of Narbonne vetch meal in the in vitro assay (Table 3). Although an addition of soluble fraction released from 50 µg of Narbonne vetch meal per unit of enzymatic activity (UA) already induced a 26.81% ± 8.92% inhibition of total protease activity, an addition of soluble fraction released from 400 µg/UA induced a 48% inhibition. Although a thermal treatment (from 60 to 100 °C) of Narbonne vetch meal, mimicking experimental feed extrusion, reduced this inhibition (Figure S1), inclusion of Narbonne vetch meal in experimental feeds still compromised total protease activity in different extents. Although an addition of 165 µg/UA of the A10 feed did not significantly induce a higher inhibition of total protease activity than the one induced by equal addition of the Control feed, higher amounts of A10 feed addition induced higher inhibition than the Control feed (ANOVA, *p* < 0.05). In contrast, addition of A30 feed as low as 165 µg/UA already induced higher inhibition of total protease activity compared to that of the Control and A10 feeds, and reaching up to a 45.86% ± 1.29% inhibition when 990 µg/UA of A30 feed was added (ANOVA, *p* < 0.05).

### 3.2. Growth Performance Indexes and Fillet Nutritional Value

During the experimental period, no mortalities occurred in the different dietary treatments and no effect on the feed intake was observed regardless of the percentage of inclusion of Narbonne vetch meal in the experimental diets (results not shown). Fish growth in terms of body wet weight (BW) and TL when fed diets containing increasing inclusion levels of Narbonne vetch differentially evolved during the 63-day trial (Figure S2). Although the 30% inclusion of Narbonne vetch meal (A30) significantly decreased fish growth at day 42 in both BW and TL terms (60.30 ± 1.21 g and 16.89 ± 0.22 cm; ANOVA, *p* < 0.05) compared to those of rainbow trout juveniles fed 0 (Control) and 10% (A10) inclusion (from 71.06 ± 4.50 to 74.84 ± 2.33 g; and from 17.88 ± 0.48 to 18.30 ± 0.27 cm), no significant differences in BW and TL were found among Control and A10 groups. At day 63, bigger differences were found in fish growth between the A30 (96.33 ± 1.03 g and 19.23 ± 0.18 cm; ANOVA, *p* < 0.05) and the rest of experimental groups. Indeed, fish growth of the Control and A10 groups were not significantly different in BW (ranging from 125.04 ± 10.27 to 137.24 ± 4.16 g) nor in TL (from 21.14 ± 0.68 cm to 21.85 ± 0.23 cm; ANOVA, *p* < 0.05).

Fish performance indexes at the end of the trial are shown in Table 4. No significant differences in the condition factor (CF) nor viscero-somatic index (VSI) were found among the three experimental groups (ANOVA, *p* > 0.05). In contrast, weight gain (WG), specific growth rate (SGR) and hepato-somatic index (HSI) were clearly reduced in the A30 group when compared to the Control and A10 groups (ANOVA, *p* < 0.05). Regarding feed conversion rate (FCR), both the A10 and A30 groups showed less efficiency (0.83 to 1.07) in converting ingested feed in fish growth than that of the fish fed the Control diet (0.78 ± 0.01; ANOVA, *p* < 0.05). Apparent protein digestibility coefficient was also only decreased when rainbow trout were fed the highest level of inclusion of Narbonne vetch meal (A30; 72.21 ± 6.15; ANOVA, *p* < 0.05) when compared to those fed the Control and A10 diets (85.11 to 93.72%). No differences in humidity, protein and fat content in fish muscle were found among the three experimental groups.

Inclusion of Narbonne vetch meal produced a significant modification on the FA profile of the rainbow trout fish fillets (Table 5). Narbonne vetch meal inclusion at 10% and 30% reduced the content of saturated FAs compared to that of the fish fed the Control diet (from 48.64% to 45.59%; ANOVA, *p* < 0.05; n = 3), increasing the percentage of total monounsaturated FAs (from 31.27% to 33.20%; ANOVA, *p* < 0.05; n = 3). Interestingly, oleic acid was significantly increased in trouts fed increasing levels of Narbonne vetch. Furthermore, although no differences in the total amount of polyunsaturated FAs were found (ranging from 20.08% to 21.31%; ANOVA, *p* > 0.05; n = 3), fish fillets from rainbow trout fed diets with inclusion of Narbonne vetch showed higher C18:3n3 values than fish fillets from the Control group, although not significantly different. Particular differences were found in the docosahexaenoic acid (DHA), decreasing in trout fed the A30 diet (0.41% ± 0.03%; ANOVA, *p* < 0.05; n = 3), but being not significantly different between trout fed the Control (0.50% ± 0.02%) and low Narbonne vetch meal inclusion (0.42% ± 0.05%; ANOVA, *p* > 0.05; n = 3) diets.

### 3.3. Histopathological Analysis

**Figure 1:**
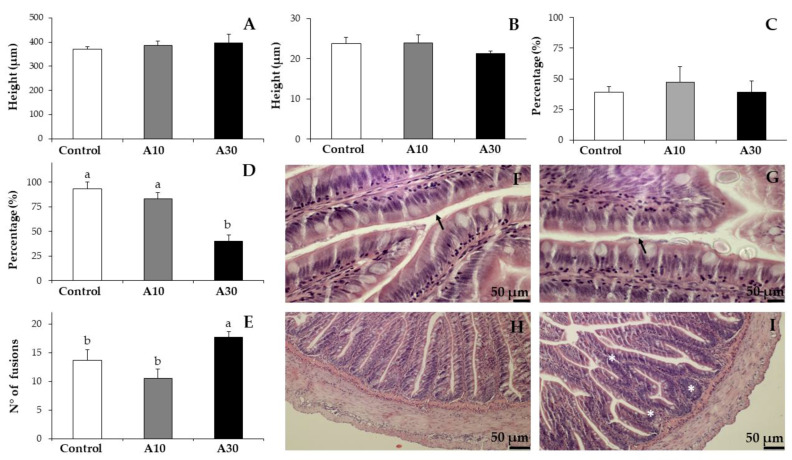
Histopathology analysis of proximal intestine showing the height of villi (**A**) and enterocytes (**B**), the percentage of supranuclear vacuolization (**C**), the percentage of brush border integrity (**D**) and the number of villi fusions per section (**E**) in rainbow trouts fed experimental diets containing increasing levels of Narbonne vetch meal: 0% (Control), 10% (A10) and 30% (A30) of inclusion. Please, note examples (arrows) of brush border integrity fully preserved (**F**) or partially degraded (**G**) and proximal intestine sections without (**H**) or showing villi fusions (asterisks; (**I**)). Different letters denote significant differences among experimental groups at each sampling day (ANOVA, *p* < 0.05; n = 3).

**Figure 2:**
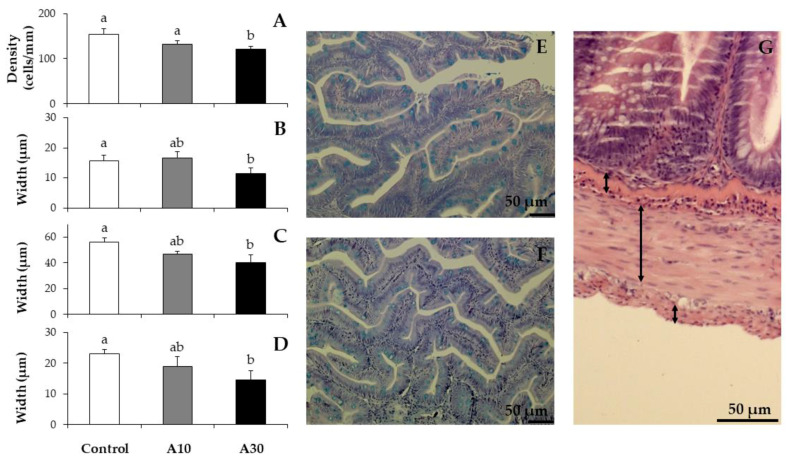
Histopathology analysis of proximal intestine showing goblet cell’s density (**A**), width of submucosa (**B**), muscular (**C**) and serosa (**D**) layers in rainbow trouts fed experimental diets containing increasing levels of Narbonne vetch meal: 0% (Control), 10% (A10) and 30% (A30) of inclusion. Please, note examples of proximal intestine sections with high (Control; (**E**)) and low (A30; (**F**)) density of goblet cells, and an example of measured width of submucosa, muscular and serosa layers (**G**). Different letters denote significant differences among experimental groups at each sampling day (ANOVA, *p* < 0.05; n = 3).

A thorough histopathological analysis was conducted to assess the impact of the inclusion of Narbonne vetch meal in rainbow trout. At the proximal intestine, neither the height of villi (from (371.28 ± 8.62 to 396.39 ± 18.41 µm), the height of enterocytes (from 21.39 ± 0.58 to 23.96 ± 2.06 µm) nor the degree of supranuclear vacuolization (from 38.89% ± 4.81% to 47.22% ± 12.73%; ANOVA, *p* > 0.05; n = 3) were altered by the inclusion of 10% or 30% of Narbonne vetch meal (Figure 1A–C). However, the highest inclusion of Narbonne vetch meal (diet A30) clearly affected the integrity of brush border (40.00% ± 6.67%) and the number of villi fusions per section (17.67 ± 1.00; ANOVA, *p* < 0.05; n = 3) when compared with the corresponding values of the A10 and Control diets (83.33 ± 5.77 and 93.33 ± 6.67%, and 10.50 ± 1.59 and 13.67 ± 1.86, respectively), which were not significantly different among them (Figure 1D–I; ANOVA, *p* > 0.05). Similarly, lower density of goblet cells, and reduced width of submucosa, muscular and serosa layers were found in rainbow trout fed the A30 diet when compared to that of trout juveniles fed with the Control diet (121.14 ± 6.84 vs. 154.49 ± 12.18 cell/mm; 11.45 ± 1.85 vs. 15.57 ± 2.06 µm; 40.05 ± 6.09 vs. 56.57 ± 2.99 µm; and 14.57 ± 3.05 vs. 23.09 ± 1.41 µm, respectively; ANOVA, *p* < 0.05). Trout juveniles fed with the A10 diet showed intermediate values, not significantly different from those of the Control group (Figure 2; ANOVA, *p* > 0.05). No significant alterations were observed regarding the position of the nucleus of the enterocytes and/or the shape and the distribution of hepatocytes, both showing a basal position and a normal shape/distribution, respectively (results not shown).

### 3.4. Blood Biochemistry Analysis

Levels of glucose, cholesterol and triglycerides in blood plasma were measured at the end of the trial (Table S1). Narbonne vetch meal inclusion did not affect the levels of these parameters in blood plasma, regardless of the dietary inclusion level. Values ranged from 72 ± 6.50 to 96.66 ± 8.56 mg/dL, from 153.55 ± 21.95 to 182.99 ± 2.74 mg/dL and from 181.54 ± 49.87 to 341.15 ± 152.68 mg/dL, respectively.

## 4. Discussion

Fish meal (FM) and fish oil (FO) have been the base of aquafeeds due to good digestibility, adequate balance of the amino acids profile and excellent palatability [49]. The increased aquaculture production during last decades in order to deal with the rise of fish consumption per capita and the constant growth of the human population, has led to an amplification of the demand to cope with animal protein requirements for food security and nutrition [1]. An enormous and tireless research effort has focused on the search for alternative raw materials to substitute FM and FO [3,4]. Although (and depending on the fish species considered) a variable (40–60%) percentage of FM can be substituted by different raw materials, mainly by SBM [50,51], the EU has placed a special interest in identifying locally produced sustainable protein sources in order to reduce the dependency on third countries as well as the carbon footprint of aquaculture products [52]. In this sense, the present study explored the safe inclusion of Narbonne vetch meal in rainbow trout diets by means of growth performance, fillet nutritional value and histopathological analysis of the digestive system. In this sense, the inclusion of other raw materials (known to affect fish physiology) was reduced to the maximum, since fish meal was the main protein source in the present feeds’ formulation.

A diverse set of alternative protein sources have been recently considered and assessed in order to partially substitute FM [31,32,33,34,35,36,37,38]. Meals of vegetable origin were largely considered as the most promising candidates [50], although several factors limited their inclusion in fish diets [9]. Among them stands the presence of different phytoestrogens and ANFs such as nonsoluble carbohydrates, lectins, saponins, nonstarch polysaccharides (NSP), phytic acid or protease inhibitors, the lower digestibility of its crude protein and/or the limited bioavailability of minerals and the deficient content in particular amino acids (e.g., lysine and methionine) [7,9,10,11,53]. Particularly, Narbonne vetch seeds is not an exception, as shown by the amino acids profile with low methionine content and as demonstrated by the in vitro inhibition of digestive protease reported here (up to a 48% reduction). Similar enzymatic activity reductions (from 30% to 50%) have been described for other fish species such as tilapia, gilthead seabream (*Sparus aurata*) or Senegalese sole (*Solea senegalensis*) when fed diets containing raw materials from vegetable origin (e.g., SBM, corn gluten or wheat bran [54]). Previous studies showed that responses to inhibition obtained when plant ingredients are incubated at different concentrations in the presence of fish digestive extracts may vary from linear to exponential [39,54]. In the present study, results obtained confirmed a similar nonlinear response when intestinal proteases of rainbow trout were tested against increased soluble fractions released from Narbonne vetch meal and experimental feeds. The presence of these ANFs greatly limits the use of these protein sources and thus, different processes have been explored to reduce their activity. In particular, exposure of those vegetable meals to high temperatures (>60 °C; such as those reached during the feed extrusion process) remove and/or decrease the activity of some ANFs [55]. The 50% reduction in the inhibitory activity of diets containing Narbonne vetch meal after thermal treatment suggested that some of the ANFs were thermolabile, such as the trypsin inhibitors [56,57]. Nevertheless, the nonthermolabile ANFs present at legume meals still reduced the protease activity, forming complexes with several minerals (e.g., calcium, magnesium and zinc, among others) that are highly required as cofactors in different enzymatic reactions [58,59,60]. In addition to GEC, Narbonne vetch was reported to contain phenolics, tannins and proteinase inhibitors (particularly trypsin and chymotrypsin inhibitors) [15,19] that might be the responsible components (at least in part) of Narbonne vetch meal inclusion reducing the reported apparent protein digestibility in rainbow trout diets rather than a potential deficiency on particular amino acids or the lower feed intake due to the presence of GEC. Since experimental feeds were formulated with enough fish meal (no partial/total substitution was performed) to warrant that any amino acid requirement for rainbow trout has been covered, an impact on growth performance of the particular amino acid profile of Narbonne vetch meal seemed to be unlikely. Furthermore, although Narbonne vetch seeds also contain GEC, which has been associated with a reduced palatability due to its sulfurous taste, reducing the feed intake in farmed monogastric animals such as in pigs [23], present strain (ZV-156) has a relatively moderate GEC content (2.89%) when compared with the previously reported values for this legume [20,61,62], in line with no effects on the feed intake regardless of the percentage of dietary inclusion in the experimental diets.

Presently, one of the major limitations of using proteins from vegetable origin in aquafeeds is the impact of their ANFs (alkaloids, glucosinolates, lectins, phytate, oligosaccharides, tannins, saponins, protease inhibitors, etc.) on the digestive system, including the reduced height of villi and enterocytes, low brush border integrity and supranuclear vacuolization in enterocytes, presence of leucocytes at lamina propria and submucosa as well as their inflammation, among other events [4,63,64,65,66,67]. In order to assess the cellular impact of Narbonne vetch meal inclusion, commonly used parameters to evaluate how nutrition affects digestive system were explored [63,64,65]. Histopathological evaluation at the proximal intestine revealed that while no negative effects were observed when a low inclusion of Narbonne vetch was performed, a 30% inclusion clearly affected the tissues and cell status of rainbow trout intestine. In contrast to the reported enteritis induced by SBM, characterized by a reduction on villi height, the presence of leucocytes at lamina propria and submucosa as well as the inflammation of these cell layers in Atlantic salmon (*Salmo salar*; [63,66,67] and rainbow trout [68], Narbonne vetch inclusion only reduced the brush border integrity and increased the incidence of fusions of villi. Therefore, Narbonne vetch meal inclusion (at least at the levels tested here, up to 30%) seemed to not induce enteritis. Indeed, the width of submucosa, muscular and serosa layers was lower than in the Control and 10% inclusion groups. Furthermore, only 30% of Narbonne vetch inclusion induced a lower density of goblet cells. Although goblet cells are well-known to secrete different mucins in order to maintain epithelial homeostasis through a mucosal barrier acting as a lubricant and helping to preserve a near-sterile epithelium, there is an increasing body of evidence showing goblet cells as a major cellular component of the innate and adaptative defense system (reviewed in [69]). In this sense, a lower density of goblet cells might indicate a lower immunocompetence in rainbow trout fed diets with 30% of Narbonne vetch inclusion. A reduced number of goblet cells were also observed in gilthead seabream when fed with diets where the 100% of FM was substituted with protein sources from vegetable origin [70]. The presence of all these histopathological alterations in the intestine has been previously related to the activity of phytate and agglutinins from soybean and other legumes that might adhere to the brush border [58,71]. Thus, a future approach to increase Narbonne vetch acceptability by fish species would be the implementation of pretreatment with phytases.

Rainbow trout growth performance values obtained here with the Control and A10 diets were within the reported range considering the initial fish size used [13,49]. Furthermore, present lower growth performance, with reduced values of SGR, WG, FCR and HSI in rainbow trout fed the highest (30%) inclusion of Narbonne vetch meal is in line with the previously published study using this vegetable protein source in tilapia [26], the related lower apparent digestibility coefficient of protein and the presence of thermostable ANFs, as well as with the histopathological conditions induced on the proximal intestine previously mentioned. Taking into account present results, where a low inclusion (10%) of Narbonne vetch meal did not reduce growth performance, this Mediterranean legume might be considered as an interesting alternative protein source for aquafeeds manufactured in the EU. Animal protein from aquatic environments used to be one of the main sources of essential FAs for human health [72,73]. In this sense, in addition to the fish growth, wellbeing and immunocompetence [74], the nutritional value of farmed fish might be compromised when FO and FM are substituted by vegetable sources. In particular, farmed fish contain important amounts of health-promoting long-chain omega-3 FAs such as eicosapentaenoic (EPA) and docosahexaenoic (DHA) acids when fed on FM- and FO-formulated diets, but those decreased when fed diets formulated with sustainable alternatives of terrestrial origin [75]. Here, the n-3/n-6 ratio to assess the nutritional value of the fillet showed values within the reported range for rainbow trout and other salmonid species, from 0.90 to 1.38 [13,76]. Food items with values higher than 1 in this ratio are considered to reduce the risk of cardiopathies, as well as inflammatory and immunological diseases [77,78,79,80]. Although no changes on the n-3/n-6 FA ratio were observed regardless of the Narbonne vetch inclusion, nor significant differences found in the content of total polyunsaturated FAs, 30% Narbonne vetch inclusion significantly decreased DHA content compared to the Control, and both 10% and 30% inclusion lead to higher amounts of monounsaturated FAs and less of the saturated ones compared to the Control diet. Variable results on FA profiles were reported, depending on the feed formulation, fish size, season and/or the temperature of the rearing water [30]. Interestingly, no alteration on the sources of oils was performed to formulate present experimental diets and thus, vegetable meal sources might alter lipid metabolism. Although lipid profile showed small differences, this unexpected result might reinforce the idea of designing future feed formulations considering not only the content of each nutrient on each raw material used, but also taking into account potential (synergistic or detrimental) interactions among them, known as the “balance strategy” [3].

The quantification of particular metabolites (e.g., glucose, cholesterol and/or triglycerides) at blood plasma has been largely used to get insights on the physiological condition of fish species in front of nutritional deficiencies/excess and/or stress conditions [81]. One of the most used is cholesterol, with its concentration reported to be decreased in plasma from different fish species when fed with an almost totally replaced FM with a mixture of plant protein sources [30,50,70,82,83]. In this case, decreased cholesterol plasma levels seem to be associated with the presence of phytoestrogens such as genistein and daidzein (abundantly found in soybean [10]), that might block its intestinal absorption [30,82,84]. In the present study, no significant differences were found in either glucose, triglycerides and/or cholesterol. While the lack of difference on plasma cholesterol levels might indicate its absorption and metabolism was not altered by Narbonne vetch meal inclusion, no differences in glucose and triglycerides might be also related to sampling blood plasma from 24-h-fasted fish. Future work to characterize the postprandial metabolism in fish fed diets containing Narbonne vetch meal needs to be performed. Nevertheless, considering that 10% inclusion of Narbonne vetch meal did not significantly affect fish growth and physiology as well as its nutritional value, although it slightly reduced the FCR compared to the one of Control diet, it can be considered as a very interesting alternative raw material to be included in sustainable and lower carbon footprint diets following the “balance strategy” due to its low price and large availability in the Mediterranean region.

## 5. Conclusions

Meal from seeds of Narbonne vetch (ZV-156 strain) can be safely included in rainbow trout diets up to 10% without affecting fish growth and performance, digestive system histopathology and/or nutritional value. Furthermore, 10% inclusion improved monounsaturated FAs. More detailed research is needed to improve its digestibility through the implementation of pretreatments to decrease the presence/activity of ANFs, and/or to elucidate the molecular pathways by which Narbonne vetch meal inclusion is limited to this low level. Longer-term nutritional trials and immune system assessment might be also required to fully characterize how fish will perform when Narbonne vetch meal is included on their diets. Nevertheless, the present results, its local production, high commercial availability and low price lead us to consider Narbonne vetch seeds as one of the most sustainable alternative raw materials to partially substitute FM and/or SBM in fish diets under the “balance strategy” approach.

## Figures and Tables

**Table 1:** Composition of Narbonne vetch (*V. narbonensis*) seeds.

**Proximate Composition (% on Dry Matter)**	
Moisture	10.9
Crude protein	30.1
Crude fat	1.18
Crude ash	3.26
Total carbohydrates	65.3
**Amino Acid (g/100 g Ingredient) Content**	
Asp	2.65
Thr	0.79
Ser	1.30
Glu	4.43
Gly	1.03
Ala	1.10
Cys	0.07
Val	0.79
Met	0.16
Ile	0.53
Leu	1.31
Tyr	0.20
Phe	0.85
His	1.09
Lys	1.63
Arg	1.39
Pro	0.95
**Antinutritional Factors (g/100 g on Dry Matter) Content**	
GEC *	2.89

* GEC: γ-glutamyl-S-ethenyl-cysteine.

**Table 2:** Ingredients and proximate composition of experimental diets.

**Ingredients (g/100 g, on Wet Basis)**	**Diets**		
	**Control**	**A10**	**A30**
Fishmeal LT *	35.9	35.9	35.9
Narbonne vetch (*Vicia narbonensis*)	0.0	10.0	30.0
Wheat gluten	12.0	11.7	12.7
Wheat meal	16.1	10.7	0.65
Soybean protein concentrate	15.5	11.2	0.0
Fish oil	13.0	13.0	13.0
Soybean lecithin	1.0	1.0	1.0
Attractant **	2.0	2.0	2.0
Vit & Min Premix	2.0	2.0	2.0
Binder (guar gum)	2.0	2.0	2.0
Methionine	0.5	0.5	0.75
**Total**	**100**	**100**	**100**
**Proximate Composition (% on Dry Matter)**			
Moisture	6.5	6.7	6.7
Crude protein	43.6	44.1	43.2
Crude fat	18.5	17.5	17.8
Crude fiber	1.25	1.38	1.56
Ash	7.6	7.1	6.7
**Antinutritional Factor (g/100 g on Dry Matter) Content**			
GEC ***	0.0	0.26	0.64

* LT stands for Low Temperature; ** fish blood meal; *** GEC: γ-glutamyl-S-ethenyl-cysteine.

**Table 3:** In vitro inhibition of rainbow trout (*O. mykiss*) digestive proteases by increasing additions of Narbonne vetch (*V. narbonensis*) meals and the experimental diets per unit of activity in 30 g body weight specimen.

**Total Protease Activity Inhibition by Narbonne Vetch Meal**			
µg Narbonne Vetch Meal/UA *		**Inhibition (%)**	
50		26.81 ± 8.92 ^b^	
100		32.17 ± 0.70 ^b^	
200		42.01 ± 4.53 ^a^	
300		46.35 ± 3.04 ^a^	
400		48.74 ± 2.14 ^a^	
**Total Protease Activity Inhibition by Experimental Feeds**			
**µg Feed/UA**	**Control**	**A10**	**A30**
165	1.65 ± 0.66 ^b^	6.24 ± 3.54 ^b^	12.01 ± 3.32 ^a^
330	3.30 ± 1.31 ^b^	14.30 ± 4.47 ^a^	17.69 ± 6.69 ^a^
495	4.96 ± 1.98 ^b^	18.52 ± 4.61 ^a^	26.99 ± 5.51 ^a^
660	6.61 ± 2.46 ^c^	20.52 ± 1.26 ^b^	37.60 ± 2.26 ^a^
990	9.91 ± 3.97 ^c^	27.47 ± 1.79 ^b^	45.86 ± 1.29 ^a^

* UA = Unit of protease activity. Different superscript letters within each column (in Narbonne vetch meal data) or row (in experimental feeds data) denote significant differences among experimental groups (ANOVA, *p* < 0.05; n = 3).

**Table 4:** Growth performance, somatic indexes, apparent protein digestibility coefficients of diets and fillet analysis in rainbow trout fed with diets containing increasing levels of Narbonne vetch meal.

**Days**	**Parameter**	**Control**	**A10**	**A30**
0	IBW (g)	26.81 ± 0.49	26.03 ± 1.29	27.46 ± 0.24
	ITL (cm)	13.59 ± 0.26	13.15 ± 0.24	13.61 ± 0.36
63	FBW (g)	137.24 ± 4.16 ^a^	125.04 ± 10.27 ^a^	96.33 ± 1.03 ^b^
	FTL (cm)	21.85 ± 0.23 ^a^	21.14 ± 0.68 ^a^	19.23 ± 0.18 ^b^
	CF	1.32 ± 0.04	1.32 ± 0.02	1.35 ± 0.02
	WG (%)	411.90 ± 10.56 ^a^	380.16 ± 24.51 ^a^	250.83 ± 2.46 ^b^
	SGR (%/day)	2.59 ± 0.03 ^a^	2.49 ± 0.08 ^a^	1.96 ± 0.01 ^b^
	FCR	0.78 ± 0.01 ^c^	0.83 ± 0.02 ^b^	1.07 ± 0.00 ^a^
	HSI (%)	1.06 ± 0.09 ^b^	1.04 ± 0.09 ^b^	1.27 ± 0.09 ^a^
	VSI (%)	11.11 ± 0.91	10.34 ± 0.27	10.36 ± 1.42
**Protein Apparent Digestibility (%)**				
ADC Protein		93.72 ± 0.39 ^a^	85.11 ± 3.83 ^a^	72.21 ± 6.15 ^b^
**Proximate Analysis of Muscle (%; on Wet Weight Basis)**				
Humidity		74.78 ± 0.67	74.99 ± 0.17	75.77 ± 0.63
Protein		17.98 ± 0.10	18.17 ± 0.34	18.12 ± 0.16
Fat		4.70 ± 0.70	4.15 ± 0.81	3.39 ± 0.43

Values are expressed as mean ± standard deviation. Different superscript letters within each row denote significant differences among experimental groups (ANOVA, *p* < 0.05; n = 3). IBW, initial body weight; ITL, initial total length; FBW, final body weight; FTL, final total length; CF, condition factor; WG, weight gain; SGR, specific growth rate; FCR, feed conversion ratio; HSI, hepato-somatic index; VSI, viscero-somatic index; ADC Protein, apparent digestibility coefficient of protein.

**Table 5:** Fatty acids profile in rainbow trout fillets when fed with diets containing increasing levels of Narbonne vetch meal.

Fatty Acid	Control	A10	A30
C12:0	0.05 ± 0.01	0.05 ± 0.01	0.05 ± 0.01
C13:0	0.08 ± 0.04	0.08 ± 0.02	0.09 ± 0.09
C14:0	4.89 ± 0.11 ^a^	4.36 ± 0.16 ^b^	4.40 ± 0.07 ^b^
C14:1	0.08 ± 0.07	0.08 ± 0.06	0.12 ± 0.07
C15:0	0.56 ± 0.02 ^a^	0.49 ± 0.02 ^b^	0.46 ± 0.01 ^b^
C15:1	0.07 ± 0.01	0.07 ± 0.01	0.08 ± 0.01
C16:0	19.76 ± 0.27 ^a^	17.69 ± 0.95 ^b^	18.09 ± 0.29 ^b^
C16:1	6.20 ± 0.13	6.02 ± 0.28	6.15 ± 0.12
C17:0	0.49 ± 0.02 ^a^	0.43 ± 0.02 ^b^	0.39 ± 0.03 ^b^
C17:1	0.39 ± 0.01 ^a^	0.37 ± 0.03 ^a,b^	0.34 ± 0.01 ^b^
C18:0	4.06 ± 0.06 ^a^	3.55 ± 0.32 ^b^	3.63 ± 0.13 ^a,b^
C18:1(n9)	15.49 ± 0.34 ^b^	18.38 ± 0.27 ^a^	18.52 ± 0.25 ^a^
C18:2(n6)	8.79 ± 0.17	9.56 ± 1.22	9.52 ± 0.41
C18:3(n3)	1.50 ± 0.03	1.61 ± 0.21	1.73 ± 0.19
C18:3(n6)	0.09 ± 0.01 ^b^	0.11 ± 0.01 ^a^	0.10 ± 0.01 ^a,b^
C20:0	0.22 ± 0.01	0.18 ± 0.01	0.18 ± 0.02
C20:1(n9)	4.36 ± 0.05 ^a^	3.95 ± 0.28 ^a,b^	3.82 ± 0.14 ^b^
C20:2	0.62 ± 0.03	0.61 ± 0.03	0.60 ± 0.04
C20:3n3	0.97 ± 0.01 ^a,b^	1.01 ± 0.02^a^	0.94 ± 0.03 ^b^
C20:3n6	0.22 ± 0.02	0.24 ± 0.02	0.23 ± 0.01
C20:4n6 (ARA)	0.2 ± 0.01	0.19 ± 0.01	0.19 ± 0.01
C20:5n3 (EPA)	7.19 ± 0.23	7.61 ± 0.31	7.36 ± 0.27
C22:0	0.15 ± 0.01	0.13 ± 0.01	0.12 ± 0.03
C22:1 (n9)	4.54 ± 0.13 ^a^	3.72 ± 0.45 ^b^	3.58 ± 0.16 ^b^
C22:6(n3) (DHA)	0.50 ± 0.02 ^a^	0.42 ± 0.05 ^a,b^	0.41 ± 0.03 ^b^
C24:0	18.36 ± 0.62	18.54 ± 0.45	18.28 ± 0.73
C24:1	0.11 ± 0.19	0.55 ± 0.58	0.59 ± 0.51
∑ Saturated	48.64 ± 0.24 ^a^	45.50 ± 1.11 ^b^	45.71 ± 0.74 ^b^
∑ Monounsaturated	31.27 ± 0.50 ^b^	33.13 ± 0.88 ^a^	33.20 ± 0.18 ^a^
∑ Polyunsaturated	20.08 ± 0.34	21.37 ± 1.79	21.09 ± 0.77
n-3/n-6	1.02	0.99	0.98

Values are expressed as % (mean ± standard deviation). Different superscript letters within each row denote significant differences among experimental groups (ANOVA, *p* < 0.05; n = 3).

## Supplementary Materials

The following are available online at https://www.mdpi.com/2076-2615/10/11/2175/s1. Figure S1: Dose-response curves of the inhibitory effect of heat-treated Narbonne vetch meal on the intestinal proteases of rainbow trout, Figure S2: Fish growth, Table S1: Serum biochemical assays in rainbow trout fed with the experimental diets including Narbonne vetch meal.
