# Supplementary material for: Effects and Safe Inclusion of Narbonne Vetch (Vicia narbonensis) in Rainbow Trout (Oncorhynchus mykiss) Diets: Towards a More Sustainable Aquaculture
Source: Animals (Basel). 2020 Nov 21;10(11):2175. doi: 10.3390/ani10112175 (PMC7700202; doi:10.3390/ani10112175)
Supplement: Supplementary file 1 [file animals-10-02175-s001.zip › Supplementary figure S1-983085.docx]

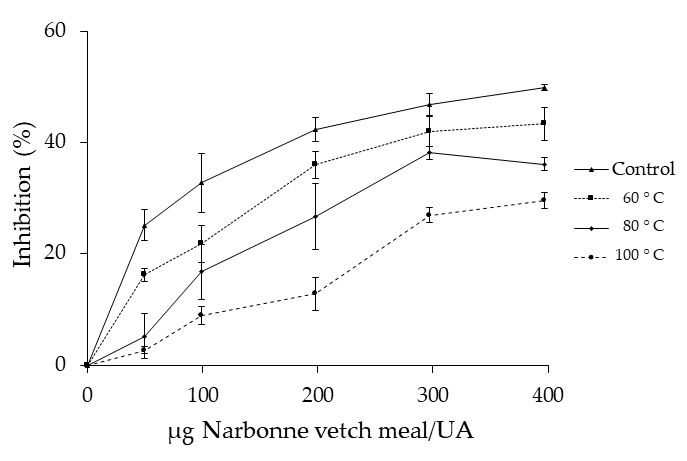


**Figure S1.** Doses-response curves of the inhibitory effect of heat-treated Narbonne vetch meal on the protease activity of rainbow trout.
