# Supplementary material for: Effects and Safe Inclusion of Narbonne Vetch (Vicia narbonensis) in Rainbow Trout (Oncorhynchus mykiss) Diets: Towards a More Sustainable Aquaculture
Source: Animals (Basel). 2020 Nov 21;10(11):2175. doi: 10.3390/ani10112175 (PMC7700202; doi:10.3390/ani10112175)
Supplement: Supplementary file 1 [file animals-10-02175-s001.zip › Supplementary figure S2-983085.docx]

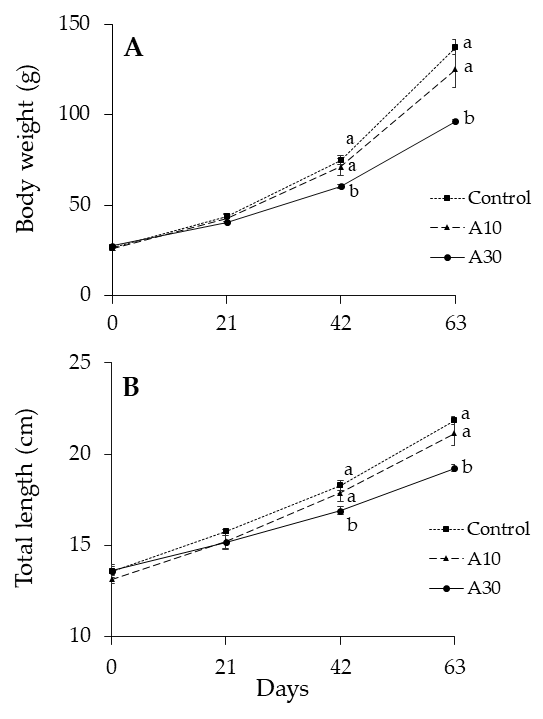


**Figure S2.** Fish growth (mean ± standard deviation) values in body weight (A) and total length (B) in rainbow trout fed experimental feeds containing increasing levels of Narbonne vetch meal: 0 (Control), 10 (A10) and 30 (A30) % of inclusion. Different letters denote significant differences among experimental groups at each sampling day (ANOVA, P < 0.05; n = 3).
