# Supplementary material for: Effects and Safe Inclusion of Narbonne Vetch (Vicia narbonensis) in Rainbow Trout (Oncorhynchus mykiss) Diets: Towards a More Sustainable Aquaculture
Source: Animals (Basel). 2020 Nov 21;10(11):2175. doi: 10.3390/ani10112175 (PMC7700202; doi:10.3390/ani10112175)
Supplement: Supplementary file 1 [file animals-10-02175-s001.zip › Supplementary table S1-983085.docx]

**Table S1.** Serum biochemical assays in rainbow trout fed with the experimental diets including Narbonne vetch meal.

| **Analysis**  **(mg/dL)** | **Diets** | | |
| --- | --- | --- | --- |
|  | **CONTROL** | **A10** | **A30** |
| Triglycerides | 341.15 ± 152.68 | 291.87 ± 56.57 | 181.54 ± 49.87 |
| Glucose | 96.66 ± 8.56 | 86.46 ± 9.72 | 81.72 ± 6.50 |
| Cholesterol | 182.99 ± 35.45 | 197.45 ± 30.81 | 153.55 ± 21.95 |
